# NLRP6 is required for cancer-derived exosome-modified macrophage M2 polarization and promotes metastasis in small cell lung cancer

**DOI:** 10.1038/s41419-022-05336-0

**Published:** 2022-10-21

**Authors:** Xinrui Rao, Xiaoshu Zhou, Geng Wang, Xiaohua Jie, Biyuan Xing, Yingzhuo Xu, Yunshang Chen, Jun Li, Kuikui Zhu, Zilong Wu, Gang Wu, Chuangyan Wu, Rui Zhou

**Affiliations:** 1 Cancer Center, Union Hospital, Tongji Medical College, Huazhong University of Science and Technology, Wuhan, 430022 China; 2 Institute of Radiation Oncology, Union Hospital, Tongji Medical College, Huazhong University of Science and Technology, Wuhan, 430022 China; 3 Department of Gastrointestinal Surgery, Union Hospital, Tongji Medical College, Huazhong University of Science and Technology, Wuhan, 430022 China; 4 Department of Thoracic Surgery, Union Hospital, Tongji Medical College, Huazhong University of Science and Technology, Wuhan, 430022 China

**Keywords:** Immunosurveillance, Cancer microenvironment

## Abstract

Metastasis remains the primary cause of small cell lung cancer (SCLC)-related deaths. Growing evidence links tumor metastasis with a pre-metastatic microenvironment characterized by an anti-inflammatory response, immunosuppression, and the presence of tumor-derived exosomes. To clarify the relationships among these factors in SCLC, we analyzed SCLC patient samples as well as a mouse model. Among the infiltrating immune cells, our study focused on the tumor-associated macrophages (TAMs), that are well-known to promote tumor progression and metastasis. We found that high expression of the alternatively activated (M2) TAM marker, CD206^+^ was associated clinically with a poorer prognosis and metastasis state in patients with SCLC. Moreover, infiltrating macrophages (MØ) were found in the metastatic foci of an SCLC mouse model. Additionally, we observed dominant switching to M2 phenotype, accompanied by increased NLRP6 expression. Since tumor-derived exosomes are the key links between the tumor and its immune microenvironment, we further investigated whether SCLC-derived exosomes contributed to the MØ phenotype switch. Our findings showed for the first time that SCLC-derived exosomes induce the M2 switch via the NLRP6/NF-κB pathway, and thus, promote SCLC metastasis in vitro and in vivo. Collectively, these results indicate a novel mechanism by which SCLC-derived exosomes induce immunosuppression of distant MØ to promote systemic metastasis by activating NLRP6. Here, we highlight the close relationship between the tumor-derived exosomes, inflammasomes and immune microenvironment in SCLC metastasis.

## Abstract (web summary)

A schematic illustration showing that SCLC-derived exosomes polarize M2 macrophages to promote SCLC metastasis through the NLRP6/NF-κB pathway.

## Graphical abstract

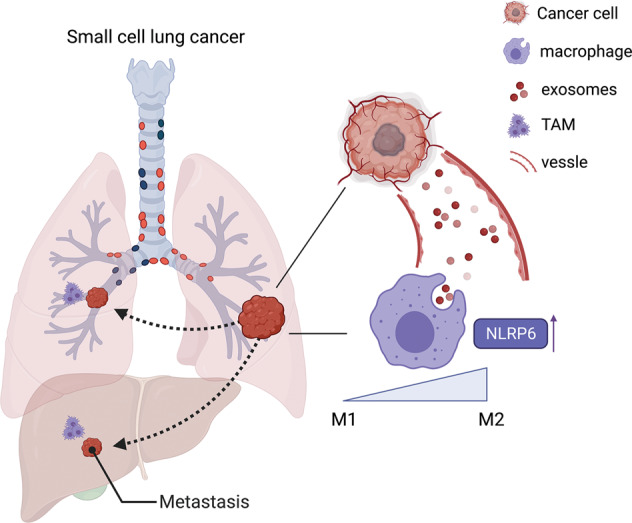

## Introduction

The major characteristic of small cell lung cancer (SCLC) is high invasiveness, which results in an extremely poor prognosis [1]. Emerging evidence has implicated immunosuppression in tumor metastasis [2, 3]. Among the tumor-infiltrating immune cells, tumor-associated macrophages (TAMs) play a pivotal role in promoting tumor progression [4]. Although the differentiation of macrophages (MØ) is heterogeneous, TAMs have historically been divided into classically activated MØ (M1), and alternatively activated MØ (M2). TAMs acquire an M2-like phenotype and resemble ‘tolerant’ MØ, which stimulate tumor progression and limit therapeutic responses. M2-like MØ have been shown to be widely involved in the cancer metastatic process, including tumor cell migration, proteolytic destruction of the matrix, and angiogenic switch [5]. Thus, these cells are crucial for enabling the spread of tumor cells [6]. Therefore, exploring the mechanisms by which TAMs promote cancer metastasis may provide new targets for the treatment of metastatic SCLC.

Cancer-promoting inflammation is now a recognized hallmark of cancer [7]. Inflammasomes are caspase-activating complexes that mediate inflammation and are involved in the regulation of cancer [8]. The nucleotide-binding oligomerization domain-like receptor family pyrin domain-containing (NLRP) proteins are a class of sentinel receptors that are pivotal for the detection of pathogenic microbes and danger signals [9]. NLRP6 belongs to the NLRP family and recruits the adapter apoptosis-associated speck-like protein (ASC) and the inflammatory caspase-1 or caspase-11 to form an inflammasome, thus regulating the maturation and secretion of the pro-inflammatory cytokines IL-18 and IL-1β. In other contexts, NLRP6 can also exert its function in an inflammasome-independent manner [10]. It can dampen the inflammatory response and acts as a counterpart to the other pattern recognition receptors (PRRs), by downregulating the TLR‐mediated NF‐κB and MAPK signaling, the pathways involved in MØ polarization [11]. Thus, NLRP6 may drive inflammatory or anti‐inflammatory functions depending on the different physiological contexts. Although it has been widely studied in intestinal injuries and malignancies as a protective factor [12], its role in SCLC metastasis remains unclear.

Cell-to-cell communication is important for the dynamic interactions between tumors and immune cells in the tumor microenvironment. Exosomes are extracellular vesicles released by various cell types and contain numerous molecular constituents of the original cells. Over the past few decades, research has revealed new facets of the role played by exosomes in cancer development and progression. Tumor cells release more exosomes compared to non-transformed cells. It has been reported that the pathological function of cancer-derived exosomes in metastasis includes the modification of immune cell response at local and distant sites [13]. Further studies have revealed that SCLC-derived exosomes can promote SCLC metastasis by delivering DNA, non-coding RNAs, proteins, and lipids. Exosomal microRNA such as miR-375-3p, miR-665, and miR-92b-3p have been demonstrated to play a role in promoting the invasion and migration of SCLC cells [14–16]. Moreover, the exosomal protein PFN2 was reported to promote proliferation, migration, and invasion of SCLC cells [17]. However, the mechanism by which SCLC-derived exosomes promote SCLC metastasis through the regulation of immune cell functions, remains poorly understood.

In this study, we newly found that M2-like MØ infiltrate SCLC metastatic foci and participate in the metastatic process. Additionally, our data newly identified that SCLC-derived exosomes induce M2 polarization via the NLRP6/NF-κB pathway in MØ cells. Inhibition of exosome secretion or NLRP6 expression reversed these effects. Moreover, SCLC-derived exosomes were found to promote SCLC metastasis in vivo. Here we elucidated the interaction between tumor-derived exosomes, inflammation components, and SCLC metastasis.

## Materials and methods

### Patient and clinical samples

Clinical samples were collected from 72 patients with SCLC who underwent CT-guided percutaneous lung biopsy or endobronchial ultrasound-guided transbronchial needle aspiration (EBUS-TBNA) at Wuhan Union Hospital, Huazhong University of Science and Technology, China. All enrolled patients were diagnosed based on the clinical pathology findings and staged by specialized oncologists according to the Veterans Administration Lung Study Group (VALG). All paraffin sections were collected in the Department of Pathology at the Wuhan Union Hospital, according to the protocols approved by the Ethics Review Board of Wuhan Union Hospital, Huazhong University of Science and Technology (S017). Informed consent was obtained from all patients involved. Clinical records were abstracted, and follow-up data were collected. All patients received standard treatments recommended by the National Comprehensive Cancer Network (NCCN) guidelines for SCLC.

### Immunohistochemistry

Immunohistochemistry assays were performed as described previously [18]. The primary antibodies were used as followed: anti-CD68 (1:100; #ab201973; Abcam), anti-CD206 (1:2000; #ab252921; Abcam), and anti-NLRP6 (1:100; #PA5-21022; Invitrogen). Three fields were randomly chosen from each slide, and the number of CD68- or CD206-positive cells were counted by two independent pathologists. NLRP6 immunohistochemistry (IHC) score was categorized as negative (1), weakly positive (2), moderately positive (3), and strongly positive (4).

### In vivo experiments

BALB/c nude mice (6 weeks old, female) were raised at the Tongji Medical College of Huazhong University of Science and Technology. All animal procedures were performed according to the National Institutes of Health Guide for the Care and Use of Laboratory Animals. Mice were randomly divided into different groups (at least five mice per group according to previous experience). To establish the SCLC tail vein-injected allograft models, ~2 × 10^6^ RT mouse SCLC cells were injected via the tail vein. To investigate the effect of SCLC-derived exosomes on tumor metastasis, exosomes (10 μg) or isopycnic PBS were injected into nude mice via the tail vein. The SCLC cells were injected 2 days later, and the mice were randomly divided into two groups which received intravenous injections of PBS (Con group), or exosomes secreted by SCLC cells (Exo group) every 2 days. All animals were sacrificed 4 weeks after the SCLC cell injection. The lungs were surgically removed from the tumor-bearing mice and the pulmonary tumors were counted. The number of tumors in the lungs was compared between the two groups.

### Flow cytometry

BMDMs, THP-1 or mouse lung cells were harvested, centrifuged, and washed with phosphate-buffered saline (PBS). Fluorochrome-labeled antibodies were added directly into the cell suspension, incubated for 1 h on ice, and the cells were washed twice in PBS. Stained cells (~10^5^ cells/sample) were analyzed by flow cytometry on a FACSCalibur system. The antibodies used were as follows: anti-mouse: Zombie Fixable Viability kit (1:100; #423106; Biolegend), aiti-CD11b (1:400; #101206; Biolegend), anti-F4/80 (1:100; #123116; Biolegend), anti-CD206 (1:100; #141706; Biolegend), anti-CD86 (1:50; #159204; Biolegend); anti-human: anti-CD206 (1:100; #321105; Biolegend), anti-CD86 (1:100; #374203; Biolegend).

### qRT-PCR

Total RNA was extracted from cells using TRIzol reagent (Invitrogen). qRT-PCR was performed using SYBR Premix Ex Taq reagents (TaKaRa) in two steps, according to the manufacturer’s instructions. The primer sequences used are listed in the Supplementary Table 1.

### Western blotting

Western blot analysis was performed according to standard procedures as previously described [19]. The antibodies used were as follows: anti-NLRP6 (1:1000; #PA5-21022; Invitrogen), anti-NLRP1 (1:1000; #PA5-116672;Invitrogen), anti-AIM2 (1:1000; #53491; CST), anti-NLRP3 (1:800; #15101; CST), anti-NLRP9 (1:1000; #ab105413; Abcam), anti-p65 (1:1000; #8242; CST), anti-phospho S536 p65 (1:1000; #3033; CST), anti-IκBα (1:1000; #ab32518; Abcam), anti-phospho S32 IκBα (1:1000; #3033; CST), and anti-β-actin (1:10000; #AC028; Abclonal). Following incubation with the primary antibody, the membranes were washed and probed with secondary antibodies (1:5000; #SA00001-1 & #SA00001-2; Proteintech) for 1 h. Proteins were visualized by chemiluminescence.

### Cell isolation and culture

The primary Rb1^flox/flox^ Trp53^flox/flox^ (RT) mouse SCLC cell was generated as described previously [20, 21] and was kindly provided by professor Hongbin Ji (Shanghai Institute of Biochemistry and Cell Biology, Chinese Academy) and preserved in the Bio-Research Innovation Center Suzhou, CAS. The human SCLC cell line H446, human acute monocytic leukemia cell line THP-1, and mouse lung epithelial cell line TC-1 were purchased from the Cell Bank of the Chinese Academy of Sciences (Shanghai, China). All cell lines were regularly tested for contamination with mycoplasma or other pathogens and authenticated using short tandem repeat (STR) profiling. The H446 and RT mouse SCLC cells were cultured in RPMI-1640 (Gibco) containing 10% fetal bovine serum. The TC-1 cells were cultured in Minimum Essential Medium (MEM, Gibco) containing 10% fetal bovine serum. The THP-1 cells were cultured in RPMI-1640 medium containing 100 ng/ml phorbol 12-myristate 13-acetate (PMA; Sigma) for 48 h to generate MØ. Bone marrow cells obtained from C57BL/6 mouse femurs were cultured in RPMI-1640 containing 10% fetal bovine serum and 20 ng/mL recombinant macrophage colony-stimulating factor (M-CSF; PeproTech) for 5 days. Following differentiation, naive mouse bone marrow-derived macrophages (BMDMs) were collected and cultured in 6-well plates.

### Non-contact co-culture

The RT mouse or human SCLC cells were seeded onto the upper chamber of a 0.4 µM polyester membrane of transwell inserts (BD Bioscience), which were placed in the wells. The BMDM or THP-1 cells were seeded onto the lower chamber of the system. After 48 h of co-culture, the two cell types were harvested for further analysis.

### Exosome isolation and inhibition

Exosomes were separated from SCLC cell cultures using the Exosome Isolation Reagent (Ribo) following the manufacturer’s instructions. First, the cell culture was collected and centrifuged (2000 × *g*, 30 min) to remove residual cells. The supernatant was transferred to a new tube to which 1/3 volume of isolation reagent was added and mixed. The mixture was refrigerated overnight at 4 °C and then centrifuged (1500 × *g*, 30 min). Sediments were collected for further analysis. The size and distribution of exosomes were verified using transmission electron microscopy (TEM; JEOL). For the inhibition of exosome release, SCLC cells were typically incubated with 15 nM of the pharmacological inhibitor, Dimethyl amiloride (DMA; Sigma) for 24 h.

### Quantification of cytokines

Culture supernatants of treated BMDMs were collected and cytokine secretion was measured by enzyme-linked immunosorbent assay (ELISA; R&D) according to the manufacturer’s instructions.

### NLRP6 knockdown by small interfering RNA (siRNA)

NLRP6 siRNA (OriGene, SR414599) and negative control siRNA transfection was performed using GenMute™ siRNA Transfection Reagent (SignaGen Laboratories) following the manufacturer’s instructions. After 48 h, NLRP6 expression levels in BMDMs were detected using qRT-PCR and western blot analysis to verify the knockdown efficacy.

### Statistical analysis

Experimental data are expressed as the mean ± standard error of mean (SEM). Statistical differences among the groups were assessed using SPSS 26.0. Patient data were expressed as average ± standard deviation (SD) for continuous variables, and count (percentage) for categorical variables. We used univariate and multivariable Cox proportional hazard regression models to explore risk factors associated with overall survival (OS). Relative risks were expressed as hazard ratios (95% confidence interval). Kaplan–Meier survival curves were plotted to explore the association between CD206 level and OS, with differences between the CD206^low^ and CD206^high^ curves compared using the log-rank test. In all experiments, categorical variables between groups were compared using the *χ*^2^-test, and continuous variables were analyzed using the Student’s *t* test (two-tailed) or analysis of variance, as appropriate. All data meet the assumptions of the tests and statistical tests are justified as appropriate. Differences were considered statistically significant at *P* < 0.05 (*P < 0.05, **P < 0.01, ***P < 0.001). All experiments were repeated at least three times.

## Results

### M2 TAMs correlate with metastasis and poor prognosis in patients with SCLC

**Fig. 1:**
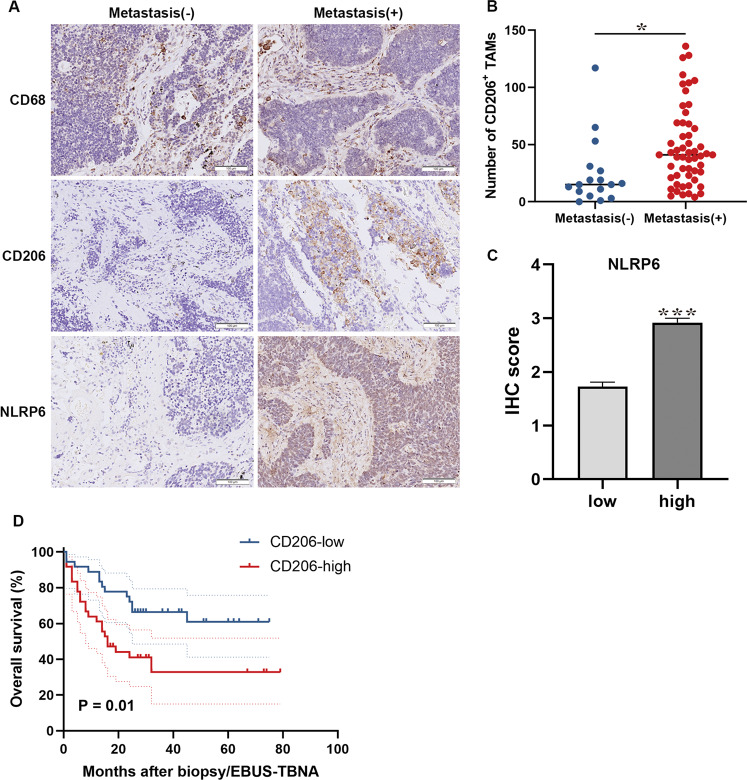
CD206+ TAMs are correlated with poor prognosis in patients with SCLC. **A** Representative images of IHC staining for CD68, CD206, and NLRP6 in primary tumors and adjacent regions in patients with SCLC. Scale bar, 100 μm. **B** Statistical analysis of the number of CD206^+^ TAMs in patients with and without metastatic SCLC (*N* = 36). **C** Statistical analysis of NLRP6 IHC score in CD206^high^ and CD206^low^ patients with SCLC (*N* = 36). **D** Survival analysis of patients with SCLC based on CD206 expression levels (*N* = 36). Bar graphs show the mean ± SEM; *P < 0.05, ***P < 0.001. TAMs tumor-associated macrophages, SCLC small cell lung cancer, IHC immunohistochemistry, SEM standard error of mean.

**Fig. 2:**
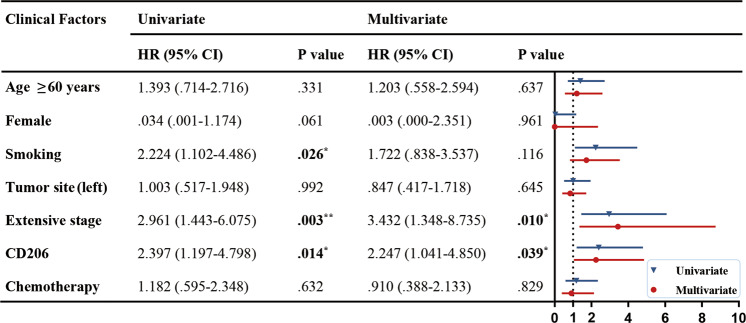
Cox proportional hazard analysis of risk factors associated with overall survival. Hazard ratios (95% confidence intervals) are shown for the risk factors associated with overall survival. Boldface type indicates statistical significance; *P < 0.05, **P < 0.01, ***P < 0.001. HR hazard ratio, CI confidence interval.

A total of 72 patients with SCLC were included in our clinical analysis (Table 1). Expression of the TAM marker CD68 and the M2 marker CD206 were analyzed in serial sections of tumor tissues from the patients. We found that CD68^+^ and CD206^+^ MØ were mainly located in the tumor stroma. No significant difference was observed in the CD68 expression levels. However, the CD206 expression was considerably higher in the patients with distant metastasis than in those without (Fig. 1A). Following this, we counted the number of CD206^+^ TAMs, which varied from 0 to 136 per field. Based on the median value, the patients were divided into two groups: low (<37 per field) and high (>37 per field) CD206^+^ TAM expression. High levels of CD206 were associated with metastasis and higher NLRP6 expression in the tumor stroma (Fig. 1B, C, Table 1). Moreover, the high CD206 expression also correlated with reduced OS (Fig. 1D). Univariate and multivariate Cox proportional hazard analyses of the data indicated that CD206 was an independent risk factor for poor prognosis (Fig. 2). Taken together, these results suggest that M2 TAMs are a risk factor for the metastasis and prognosis of SCLC.

**Table 1:** Correlation between the CD206^+^ TAMs and clinical parameters (*n* = 72).

Parameters	No. (%)	CD206 expression		
	All	low	high	^a^*P*
**Age**	59 ± 9.11	57 ± 8.56	61 ± 9.45	0.121
**Female**	11 (15.3%)	5 (13.9%)	6 (16.7%)	0.743
**Smoking**	17 (23.6%)	9 (25%)	8 (22.2%)	0.781
**Tumor site**				
Left lung	35 (48.6%)	18 (50%)	17 (47.2%)	0.814
Right lung	37 (51.4%)	18 (50%)	16 (52.8%)	
**Tumor size, cm**				
≥5	27 (37.9%)	12 (33.3%)	15 (41.7%)	0.523
<5	45 (62.1%)	24 (66.7%)	21 (58.3%)	
**Tumor stage**				
Limited stage	59 (81.9%)	31 (86.1%)	28 (77.8%)	0.358
Extensive stage	13 (18.1%)	5 (13.9%)	8 (22.2%)	
**Metastasis**	56 (77.8%)	24 (66.7%)	32 (88.9%)	0.023^*^
**Overall**	72 (100%)	36 (50%)	36 (50%)	

Data were expressed as average ± standard deviation (SD) for continuous variables and count (percentage) for categorical variables. Boldface type indicates statistical significance.

^a^*P*-values indicate differences between CD206-high and CD206-low expressed patients.

*P < 0.05.

### NLRP6 expression is elevated in metastatic foci in an SCLC nude mouse model

**Fig. 3:**
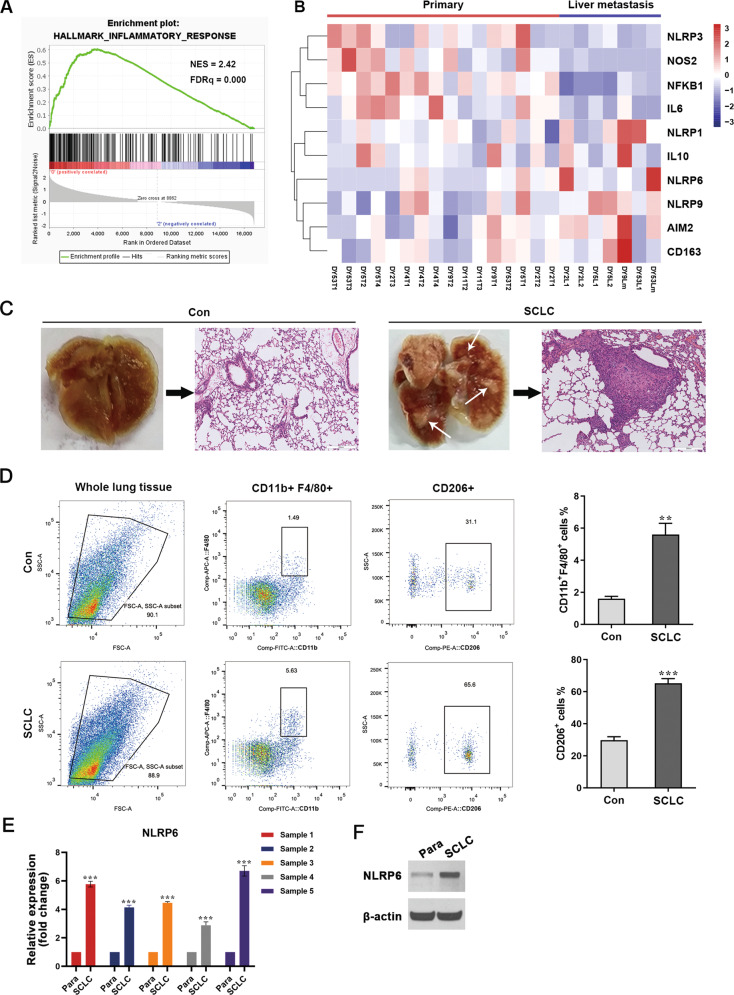
M2 TAMs infiltrate into pulmonary SCLC site. **A** GSEA plots showing the enrichment of the inflammatory response pathway in the primary site. **B** A heatmap showing expression levels of genes associated with the inflammatory response in primary tumors versus liver metastasis. **C** Lungs were excised from tumor-bearing mice and pulmonary SCLC tumors were isolated. Gross specimens and H&E-stained images of lungs are shown. White arrows point to the SCLC site. Con, untreated control mice. SCLC, mice injected with SCLC cells through tail vein. **D** Flow cytometry analysis of the polarization state of infiltrated macrophages in the lung tumor site of the SCLC nude mouse allograft model (*n* = 5). **E** qRT-PCR analysis of NLRP6 expression in lung tumor site from the SCLC nude mouse allograft model (*n* = 5). **F** Immunoblot analysis of NLRP6 expression in lung tumor site from the SCLC nude mouse allograft model (*n* = 5). Para, para-cancerous tissue. SCLC, SCLC cancerous tissue. Bar graphs show the mean ± SEM; **P < 0.01, ***P < 0.001. TAMs tumor-associated macrophages, SCLC small cell lung cancer, GSEA gene set enrichment analysis, H&E hematoxylin and eosin, SEM standard error of mean.

The inflammatory microenvironment is a key element in the immune regulation of malignant cancer. We investigated the Gene Expression Omnibus (GEO) and analyzed GSE116977, an RNA-seq dataset of primary tumors and metastasis from an Rb1^flox/flox^; Trp53^flox/flox^; p130^flox/flox^; R26^mTmG^ SCLC mouse model. The model was constructed previously via the intratracheal delivery of an adenoviral vector expressing Cre recombinase under the control of the broadly expressed cytomegalovirus (CMV) promoter [22]. The gene set enrichment analysis (GSEA) revealed that the inflammatory response pathway was significantly enriched in the primary site compared to the metastatic site (Fig. 3A), thereby indicating immunosuppression at the metastatic site. Moreover, several inflammasomes, including NLRP6, showed different expression levels between primary tumors and liver metastatic tumors. Additionally, the M2 MØ markers CD163 and IL10 were upregulated in metastatic sites, whereas the M1 markers, NOS2 and IL6 were downregulated (Fig. 3B). Though the differences in the expression levels of some genes were not statistically significant (Fig. S1), it suggested that inflammasomes and TAMs may be involved in SCLC metastasis. To validate these data, we analyzed the pulmonary tumors isolated from the RT SCLC tail vein-injected allograft model (Fig. 3C). The polarization state of infiltrated MØ was evaluated using flow cytometry. The results indicated the presence of a higher percentage of both total and M2 MØ in mice injected with SCLC cells compared with those without, consistent with the above clinical data (Fig. 3D). To estimate the changes in the levels of the inflammasome proteins in the SCLC site, qRT-PCR and western blotting analyses of AIM2, NLRP1, NLRP3, NLRP6, and NLRP9 were performed. Surprisingly, the expression of NLRP6 was significantly upregulated in the cancerous tissue compared to that in the para-cancerous tissue (Figs. 3E, F and S2). These results suggest that NLRP6 may be closely correlated with MØ polarization and metastasis in SCLC.

### SCLC cells modulate the M2 polarization of BMDMs via exosome secretion

**Fig. 4:**
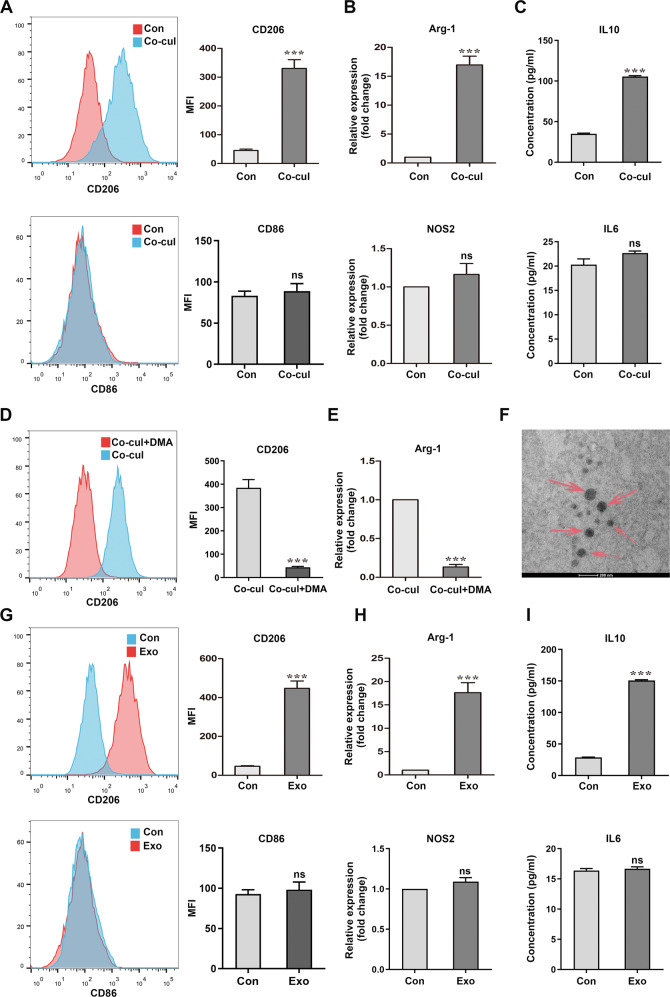
SCLC cells promote the M2 polarization of BMDMs by secreting exosomes. **A** Flow cytometry of the expression levels of the M2 polarization marker CD206 and M1 polarization marker CD86 in BMDMs without or with RT SCLC cell co-culture (*n* = 3). **B** qRT-PCR analyses of the expression levels of the M2 polarization marker Arg-1 and M1 polarization marker NOS2 in BMDMs (*n* = 3). **C** ELISA results of IL10 and IL6 in supernatant of BMDMs (*n* = 3). **D**, **E** Analysis of BMDMs, co-cultured with SCLCs post DMA treatment. **D** Flow cytometry of the expression levels of CD206 in BMDMs in a co-culture system with or without DMA treatment (*n* = 3). **E** qRT-PCR analyses of the expression levels of Arg-1 in BMDMs (*n* = 3). **F** A TEM image of exosomes isolated from RT SCLC cell culture medium. Scale bar, 200 nm. **G**–**I** Analysis of BMDMs cultured with SCLC-derived exosomes. **G** Flow cytometry of the expression levels of CD206 and CD86 in BMDMs treated without or with SCLC-derived exosomes (*n* = 3). (H) qRT-PCR analyses of the expression levels of Arg-1 and NOS2 in BMDMs (*n* = 3). **I** ELISA results of IL10 and IL6 in supernatant of BMDMs (*n* = 3). Con, untreated BMDMs. Co-cul, BMDMs co-cultured with RT SCLC cells. Exo, BMDMs co-cultured with SCLC-derived exosomes. Bar graphs show the mean ± SEM; ***P < 0.001. SCLC small cell lung cancer, BMDMs bone marrow-derived macrophages, DMA dimethyl amiloride, TEM transmission electron microscopy, SEM standard error of mean.

To determine whether MØ polarization was induced by SCLC cells, we co-cultured BMDMs and mouse RT SCLC cells. After 48 h of co-incubation with SCLC cells, the BMDMs were collected for analysis (Fig. S3). Flow cytometry and qRT-PCR analyses revealed a significantly elevated expression of the M2 markers, arginase-1 (Arg-1) and CD206. In contrast, no significant alteration was found in the expression of the M1 markers, CD86 and NOS2. These results indicated an MØ-to-M2 phenotype switch in the co-cultured BMDMs. In addition, they expressed higher levels of the M2 marker IL10, but not of the M1 marker IL6 (Fig. 4A–C). Similar results were obtained in THP-1 cells co-cultured with H446, which is a human SCLC cell line (Fig. S4). These results suggest that SCLC cells induce MØ into an immune-suppressed state.

We then investigated the mechanism by which the SCLC cells affected BMDMs without direct contact. Since SCLC-derived exosomes may be the key element connecting tumor cells with immune cells, we treated SCLC cells in the transwell system with DMA, a pharmacological inhibitor of exosome release. Analysis of these cells revealed a reversal of the MØ phenotype switch to M2, in BMDMs after DMA treatment (Fig. 4D, E). To ascertain that the tumor-derived exosomes were responsible for the observed phenotype switch, exosomes isolated from the culture media of SCLC cells (Fig. 4F) were added to BMDMs and co-cultured for 48 h. The results of flow cytometry, qRT-PCR, and ELISA experiments performed for the markers mentioned above, revealed that the exosomes from SCLC were sufficient to modify BMDMs into the M2 phenotype (Fig. 4G–I). Thus, these data suggest that SCLC-derived exosomes polarize the MØ population into the Arg-1^+^CD206^+^ M2, similar to TAMs.

### Regulation of TAM polarization by SCLC-derived exosomes is dependent on the NLRP6/NF-κB pathway

**Fig. 5:**
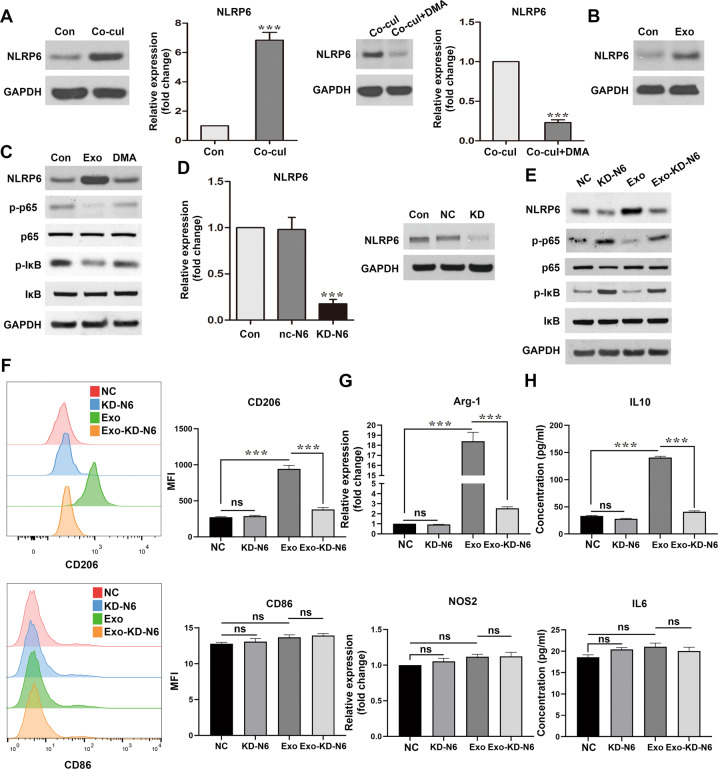
NLRP6 is required for macrophage phenotype switch. **A** Immunoblot and qRT-PCR analyses of NLRP6 expression in BMDMs co-cultured with RT SCLC cells with or without DMA treatment (*n* = 3). **B** Immunoblot analyses of NLRP6 expression in BMDMs co-cultured with SCLC-derived exosomes (*n* = 3). **C** Immunoblot analysis of IκBα, p-IκBα, p65, and p-p65 expression in BMDMs, after culturing with exosomes with or without DMA treatment of the SCLC cells (*n* = 3). **D** qRT-PCR and immunoblot analyses to confirm the knockdown efficiency in NLRP6-silenced BMDMs. **E** Immunoblot analysis of NLRP6, IκBα, p-IκBα, p65, and p-p65 expression (*n* = 3). **F** Flow cytometry of the expression levels of the M2 polarization marker CD206 and M1 polarization marker CD86 in BMDMs after culturing with exosomes in BMDMs with or without NLRP6 silencing (*n* = 3). **G** qRT-PCR analyses of the expression levels of the M2 polarization marker Arg-1 and M1 polarization marker NOS2 in BMDMs (*n* = 3). **H** ELISA results of IL10 and IL6 in the supernatant of BMDMs (*n* = 3). Con, untreated BMDMs. NC, BMDMs transfected with negative control siRNA. KD-N6, BMDMs transfected with NLRP6 siRNA. Exo, BMDMs co-cultured with SCLC-derived exosomes. Exo-KD-N6, BMDMs with NLRP6 silencing and co-cultured with SCLC-derived exosomes. Bar graphs show the mean ± SEM; ***P < 0.001. BMDMs bone marrow-derived macrophages, SCLC small cell lung cancer, DMA dimethyl amiloride, SEM standard error of mean.

NLRP6 has previously been demonstrated to inhibit the inflammatory response of cells, based on the physiological context. Considering the above findings, we hypothesized that SCLC-derived exosomes promote TAM polarization through the NLRP6 pathway. qRT-PCR and western blotting were performed to estimate the changes in NLRP6 expression in BMDMs following their co-culturing. Unsurprisingly, NLRP6 expression was significantly elevated in BMDMs after the co-culture with RT SCLC cells or exosomes directly. Meanwhile, this phenotype was reversed by DMA incubation (Fig. 5A, B). Furthermore, NLRP6 expression was increased in THP-1 cells after their co-culture with H446 (Fig. S5). Since NLRP6 negatively regulates the NF-κB signaling pathway [23, 24], we tested the p65 and IκB levels in the co-cultured BMDMs. While the total p65 and IκB levels remained unchanged, the phospho-p65 and phospho-IκB levels significantly decreased, indicating that NLRP6 might negatively regulate NF-κB activation in this system. DMA treatment prior to exosome extraction reversed the effect on phospho-IκB and phospho-p65 (Fig. 5C). Therefore, we concluded that NF-κB is a critical downstream factor of NLRP6 in SCLC tumor-derived exosome-induced MØ polarization.

To further investigate the correlation between the tumor-derived exosomes, NLRP6 expression, and the MØ phenotype switch, NLRP6 expression was knocked down in BMDMs by siRNA silencing. The transfection efficiency was analyzed using western blotting and qRT-PCR (Fig. 5D). Consistent with the above results, knockdown of NLRP6 promoted the phosphorylation of IκB and p65 (Fig. 5E). Furthermore, the results of flow cytometry and qRT-PCR analysis of the M2 and M1 markers, and ELISA analyses of the IL10 and IL6 cytokines revealed that BMDMs failed to switch to the M2 phenotype in the knockdown group following SCLC-derived exosome treatment (Fig. 5F–H). Knockdown and negative control groups also had comparable levels of the M1 markers (Fig. 5F–H). Altogether, these results support the idea that SCLC-derived exosomes promote the M2 switch in MØ through NLRP6/NF-κB pathway.

In order to investigate the contents of the exosomes that induce M2 phenotype switch, we focused on PPAR‐γ and TNF‐α, which were previously revealed to induce the transcription of NLRP6 [10]. Thus, we examined the expression level of PPAR‐γ and TNF‐α in SCLC-derived exosomes. Both the proteins were overexpressed in the SCLC- derived exosomes compared to exosomes released by the normal lung epitheliums (Fig. S6). These data implied that NLRP6 activation by SCLC-derived exosomes may not depend on one single factor, but results from the interaction of multiple factors.

### SCLC-derived exosomes promote metastasis in vivo

**Fig. 6:**
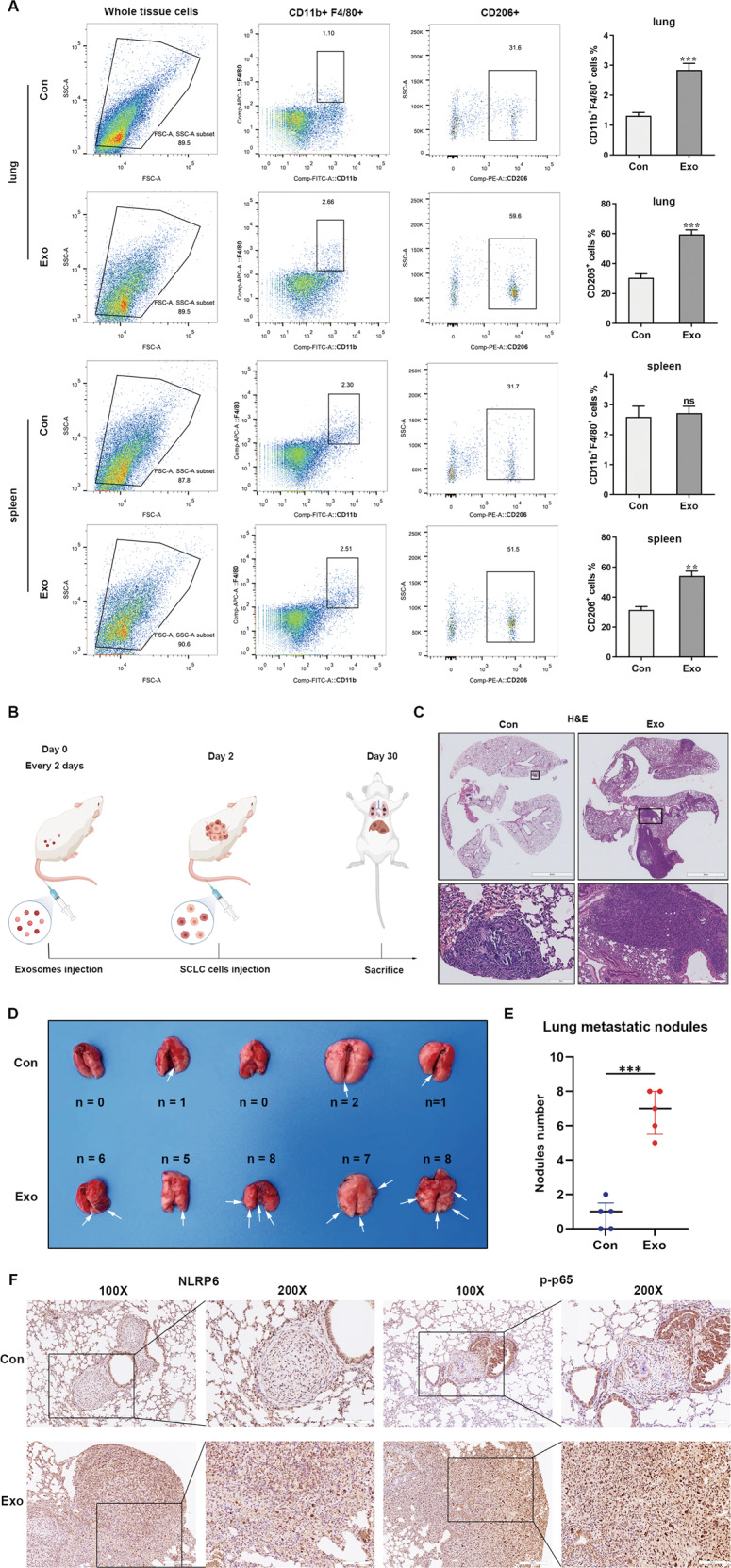
SCLC-derived exosomes promote metastasis in vivo. **A** Flow cytometry analysis showing that SCLC-derived exosomes promote M2 macrophages switch in vivo (*n* = 5). Con, mice treated with PBS. Exo, mice treated with exosomes. **B** A schematic representation of the in vivo study design and experimental workflow. **C** SCLC-derived exosomes increased lung SCLC tumor number in vivo. White arrows point to the SCLC tumor site (*n* = 5). Con, SCLC tail vein-injected mouse models treated with PBS. Exo, SCLC tail vein-injected mouse models treated with exosomes. **D** Representative images of H&E staining in SCLC tumor site in different groups. **E** Statistical analysis of the number of visible lung tumors in different groups (*n* = 5). **F** Representative IHC images of NLRP6 and p-p65 in SCLC tumor site in different groups (*n* = 5). Bar graphs show the means ± SEM; **P < 0.01, ***P < 0.001. SCLC small cell lung cancer, H&E hematoxylin and eosin, IHC immunohistochemistry, SEM standard error of mean.

To further validate the effect of SCLC-derived exosomes on M2 MØ polarization in vivo, flow cytometry analysis was performed 1 week following exosome injections into nude mice. Compared with those in the control group injected with PBS, the exosome-treated mice had a significantly higher percentage of CD206^+^ MØ in the lung and spleen (Fig. 6A). To determine whether SCLC-derived exosomes promote metastasis in vivo, exosomes isolated from mouse RT SCLC cells were injected intravenously into an SCLC tail vein-injected allograft model (Fig. 6B). After 4 weeks, the number of pulmonary tumors was counted. Exosome-treated group displayed a significantly higher number of pulmonary SCLC tumors than the control group. Moreover, immunohistochemistry revealed that NLRP6 and phospho-p65 levels were increased in exosome-treated group compared to control group (Fig. 6C–F).

Thus, our results revealed that a high proportion of M2 TAMs in SCLC tumors is related to the metastasis and poorer survival of patients with SCLC. SCLC-derived exosomes are responsible for the M2 polarization of TAM via the NLRP6/NF-kB signaling pathway, and consequently promote SCLC metastasis. Collectively, our study sheds further light on the connection between TAMs and SCLC cells in the tumor microenvironment, in which inflammasomes play crucial roles in SCLC metastasis.

## Discussion

Although the invasiveness and metastasis of SCLC are known to seriously affect patient prognosis, their specific mechanisms have not been fully elucidated, causing treatment efficacy to remain unsatisfactory [25]. Therefore, research into the mechanisms of SCLC metastasis and identification of new therapeutic targets could aid the prevention and treatment of SCLC. Here, we provide new insights into the relationship between exosomes and MØ education in the SCLC metastatic process.

Accumulating evidence has demonstrated that immunomodulation is a critical factor for the formation of a distant metastatic microenvironment [26]. In addition, the polarization state of infiltrating MØ has been shown to play a prominent role in this process. Alternative activation of MØ is associated with multiple malignant processes, including the promotion of cancer cell invasion, angiogenesis, matrix remodeling, and adaptive immunity suppression [27]. It has been reported that M2 MØ polarization promotes metastasis in multiple kinds of malignances such as colorectal cancer, pancreatic cancer, and gastric cancer [28–30]. In non-small cell lung cancer (NSCLC), M2 TAMs promote cancer cell growth and migration [31, 32]. Until recently, there has been no report on the association between MØ education and SCLC metastasis. In this study, we revealed that higher number of M2 TAMs correlated with metastasis and poorer prognosis in patients with SCLC. Consistent with the clinical data, MØ in the lung from SCLC mouse allograft model predominantly displayed the M2 phenotype, suggesting a role for the immunosuppression of MØ in facilitating SCLC invasion. It must be further noted here that in our study, we used an SCLC tail vein-injected allograft model to understand SCLC metastasis. However, this approach has limitations that include the lack of a primary tumor, regional lymph node, and other distant metastases apart from within the lung. Using a genetically engineered RT SCLC mouse model may better mimic the metastatic process of SCLC and can be tested by future research.

Most infiltrating MØs differentiate from circulating monocytes rather than from resident MØ or migrate from the primary tumor site [6]. MØ modification must require cell-to-cell communication. Classic cell-to-cell communication is mainly regulated via specific junctions, direct adhesion, and soluble growth factors. However, previous studies have indicated that the mechanism of educating the MØ remotely is mainly mediated by the exosomes derived from tumor cells [33, 34]. In agreement with these studies, we showed that exosomes released from SCLC cells could switch MØ into an M2 phenotype. Since this switch in other tissues initiates the process of metastasis [35], the induction of this phenotype by SCLC-derived exosomes could partially explain how exosomes promote distant metastasis in SCLC in vivo. The cellular origin of exosomes can be inferred based on their content, which includes nucleic acids, proteins, lipids, amino acids, and metabolites [36]. NLRP6 expression is reported to be regulated at the transcriptional and post‐translational level by metabolic stimuli and other inflammatory signals such as PPAR‐γ and TNF‐α [10]. It is noteworthy that we found a higher expression level of these proteins in SCLC-derived exosomes, and these might serve as the potential molecular triggers to switch MØ phenotype by exosomes. NLRP6 promoter region has been reported to contain binding sites for the transcription factor PPAR‐γ. Not only can PPAR‐γ be packaged into exosomes but also its overexpression increases NLRP6 mRNA transcription [10, 37, 38]. TNF‐α is also an exosome transported cytokine, that induces the transcription of NLRP6 [10, 39, 40]. Despite these findings, the precise molecular mechanism of SCLC-derived exosome induction is more complex and further analyses are required for its investigation in the future.

The inflammatory microenvironment has recently attracted the attention of researchers, as it balances the immune-suppressive and cytotoxic responses of the cells and thereby influences lung cancer outcomes [41]. The NLRP protein family, which is a component of the inflammasome, forms a complex with ASC to recruit and activate caspase-1, IL-1, and IL-18 [42]. Activation of NLRP3 has been reported to induce IL-1β signaling in dendritic cells, leading to immune activation against tumors [43]. Previously, Yang et al. showed that NLRP3 is a potential biomarker for lung adenocarcinoma progression and metastasis [44]. NLRP6 is a relatively less documented member of the NLRP family. To date, it is the only member known to inhibit inflammation. Studies have unveiled that NLRP6 shows protective effects after a peripheral nerve injury, independent of inflammasomes [45]. In intestinal epithelial cells, the NLRP6 inflammasome is suggested to play a critical role in regulating gut microbiome composition, goblet cell function and related susceptibility to gastrointestinal inflammatory, infectious and neoplastic diseases. NLRP6 protects against inflammation-associated colon tumorigenesis through repression of IL‐6 signaling [46]. While NLRP6 has been shown to suppress the tumorigenicity of gastric cancer and colorectal cancer [47], we found that it contributes to the metastasis of SCLC. In our study, we analyzed the expression of different inflammasome proteins in SCLC sites from the tail vein-injected SCLC mouse model and found that the increase of NLRP6 expression was the highest among other proteins. Furthermore, our studies on the role of SCLC-derived exosomes in the activation of NLRP6 expression indicated that inhibiting the exosome release significantly represses the elevation in NLRP6 expression as well as the MØ M2 switching. Knocking down NLRP6 expression in BMDM reversed the exosome-induced MØ switching, suggesting that NLRP6 is the key protein responsible for the education of MØ by exosome stimulation. More novel inflammasome proteins should be studied and their functions investigated in SCLC in the future.

It has been reported that NLRP6 is involved in the negative regulation of common intracellular signaling pathways, such as the NF-κB and MAPK, independent of the inflammasome [48]. Prior studies have shown that tumor-derived exosomes are a novel factor promoting M2 polarization via the NF-κB signaling pathway in melanoma and breast cancer [49, 50]. Our study brings these two aspects together, by showing for the first time that the SCLC-derived exosomes enhance NLRP6 expression and thereby inhibit both IκB and p65 phosphorylation, to promote M2 polarization. The NF-κB family of transcription factors has an essential role in inflammation and innate immunity and participates in the multiple-step process of cancer such as cell proliferation, apoptosis, angiogenesis, and metastasis [51]. IκB acts as a negative regulator of NF-κB-dependent transcription, preventing the release of the p65 subunit of NF-κB, thus limiting inflammation in the absence of further signaling events. Moreover, decreased NF-κB phospho-p65 also results in the M2 polarization of MØ [23].

In conclusion, our research revealed the relationship between SCLC-derived exosomes and the MØ phenotype switching, which involved the activation of the NLRP6/ NF-κB pathway, thus promoting SCLC metastasis in vivo. This study presents a previously unrecognized exosome-mediated pathway of MØ immunomodulation in SCLC and broadens our understanding of the regulatory mechanisms of inflammatory factors and metastasis. Further studies should focus on the identity of the specific molecules transferred from SCLC-derived exosomes to circulating MØ, including proteins, mRNAs, ncRNAs, or heredity genomic DNA. In addition, other newly discovered inflammasomes should be studied in SCLC, to provide more potential targets for clinical use. Overall, this research on inflammatory factors may offer a new direction in preventing the metastasis of SCLC.

## Supplementary information

supplementary material

Original Data File-WB

aj-checklist
