# Supplementary material for: NLRP6 is required for cancer-derived exosome-modified macrophage M2 polarization and promotes metastasis in small cell lung cancer
Source: Cell Death Dis. 2022 Oct 21;13(10):891. doi: 10.1038/s41419-022-05336-0 (PMC9587220; doi:10.1038/s41419-022-05336-0)
Supplement: Supplementary file 1 — supplementary material [file 41419_2022_5336_MOESM1_ESM.docx]

**Supplementary Table 1. The primer sequences used in qRT-PCR**

| **Gene** | **Forward 5’ to 3’** | **Reverse 3’ to 5’** |
| --- | --- | --- |
| **Mouse** |  |  |
| NLRP1 | ACTTGCTTCGTACGTGCTCC | AAGTGGCTCAGCATGAAGGT |
| NLRP3 | TGTGTGGATCTTTGCTGCG | GGAATGTGATGTACACGTGTCATTG |
| NLRP6 | CCAGGTGAAGACACTCAGGA | TGAGGGTCTTTAGGGAGCATT |
| AIM2 | TTGTCTCCTTCCTCGCACTT | GGAACAATTGTGAATGGGCT |
| NLRP9 | AGATCTCGGCTGTCCTGACT | AGGCCAGGTCTTCACAACAG |
| Arg-1 | TGTCCCTAATGACAGCTCCTT | GCATCCACCCAAATGACACAT |
| NOS2 | ACATCGACCCGTCCACAGTAT | CAGAGGGGTAGGCTTGTCTC |
| GAPDH | AGGTCGGTGTGAACGGATTTG | GGGGTCGTTGATGGCAACA |
| **Human** |  |  |
| Arg-1 | GTGGAAACTTGCATGGACAAC | AATCCTGGCACATCGGGAATC |
| NOS2 | TTCAGTATCACAACCTCAGCAAG | TGGACCTGCAAGTTAAAATCCC |
| GAPDH | GGAGCGAGATCCCTCCAAAAT | GGCTGTTGTCATACTTCTCATGG |

**Supplementary Figure 1. Statistical analysis of mRNA expression level of inflammasomes and MØ polarization markers between primary tumors and liver metastatic tumors from SCLC mouse model.** Pri, primary tumors. Lm, liver metastatic tumors. Bar graphs show the means ± SEM. ** P<0.01.*** P<0.001. SEM: standard error of mean; ns: not significant.

**

**

**Supplementary Figure 2.** **Inflammasomes except NLRP6 are not upregulated in macrophages from metastatic foci of an SCLC nude mouse model.** (A) qRT-PCR results for typical inflammasome-associated molecules, such as AIM2, NLRP1, NLRP3 and NLRP9 in lung tumor site from the SCLC nude mouse xenograft model. (B) Immunoblot results for AIM2, NLRP1, NLRP3 and NLRP9 in lung tumor site from the SCLC nude mouse allograft model. Para, para-cancerous tissue. SCLC, SCLC cancerous tissue. Bar graphs show the mean ± SEM. SEM: standard error of mean; ns: not significant.


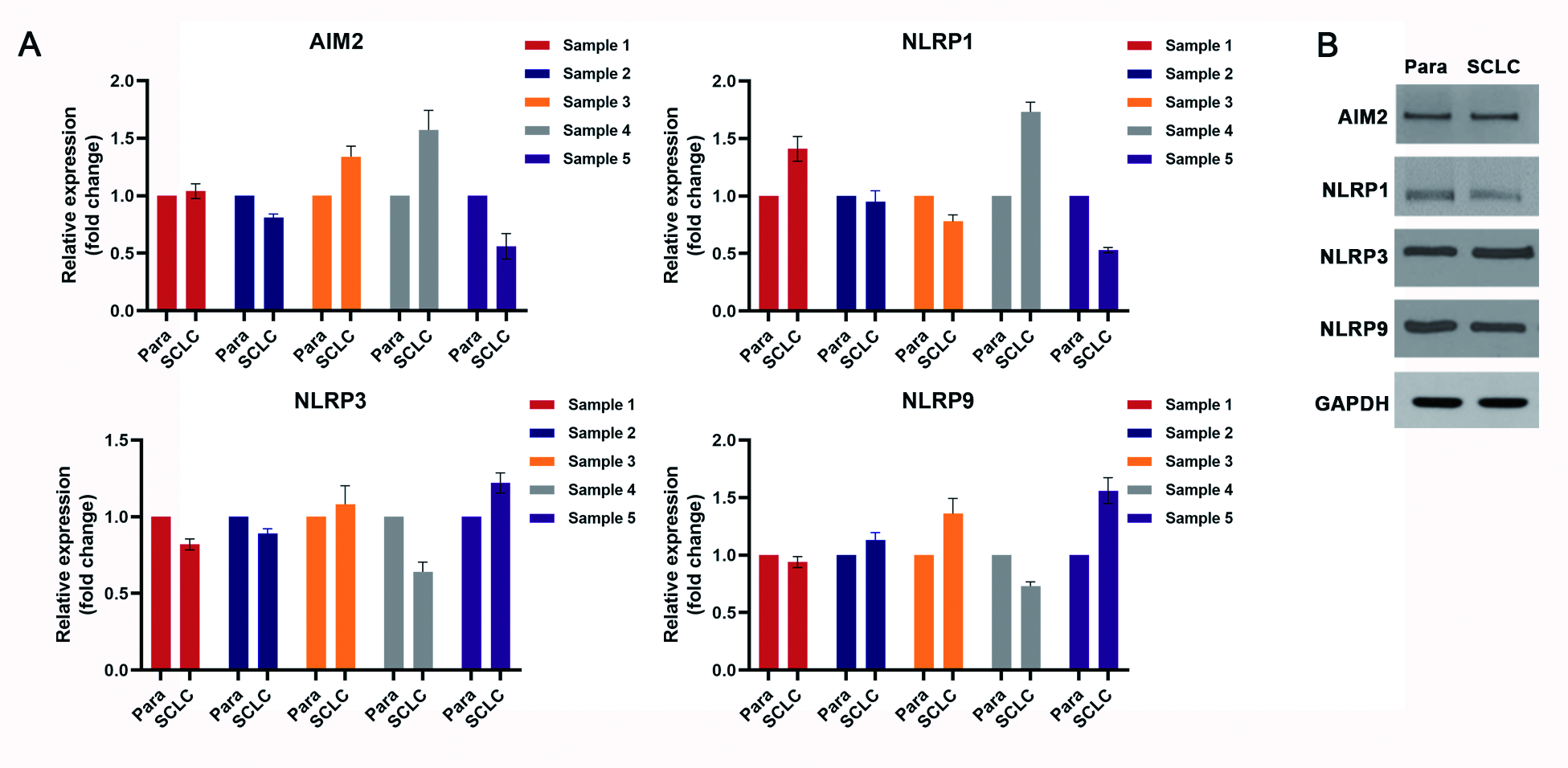


**Supplementary Figure 3.** **A schematic representation of the co-culture study design and experimental workflow.**


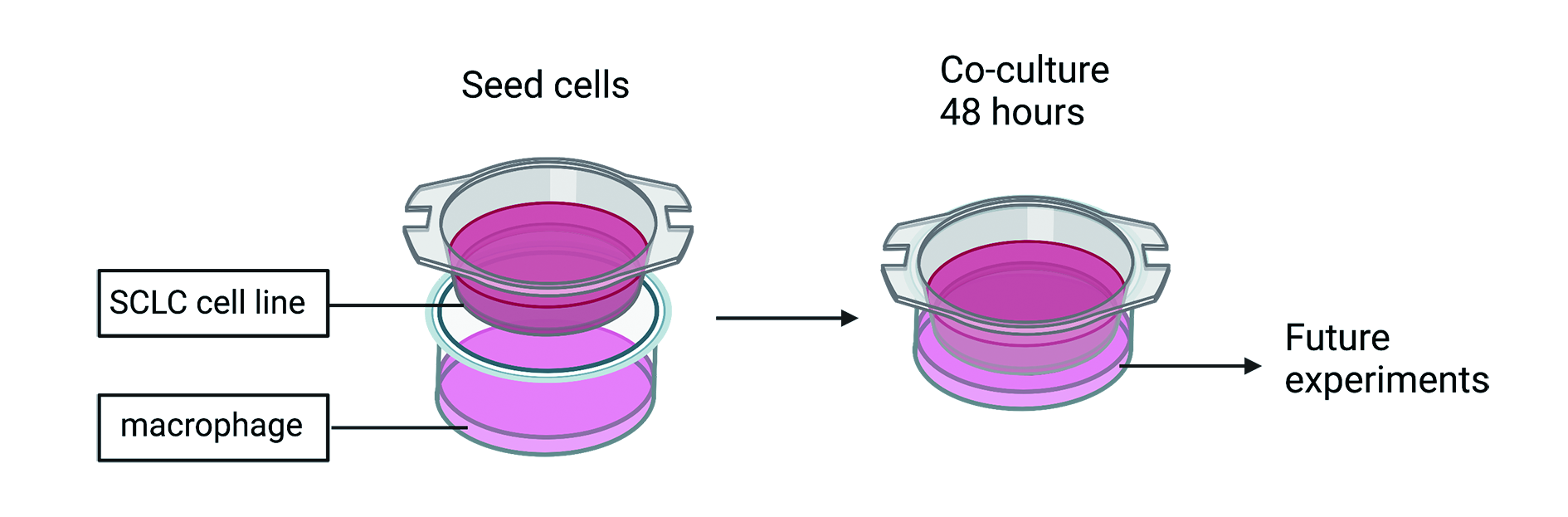


**Supplementary Figure 4. H446 promoted M2 polarization of THP-1.** (A) The expression levels of the M2 polarization markers CD206 and the M1 polarization markers CD86 by flow cytometry in THP-1 with or without human SCLC cell line H446 co-culture (n = 3). (B) The expression levels of the M2 polarization markers Arg-1 and the M1 polarization markers NOS2 by qRT-PCR in THP-1 with or without H446 co-culture (n = 3). Bar graphs show the means ± SEM; *** P<0.001. SEM: standard error of mean; ns: not significant.


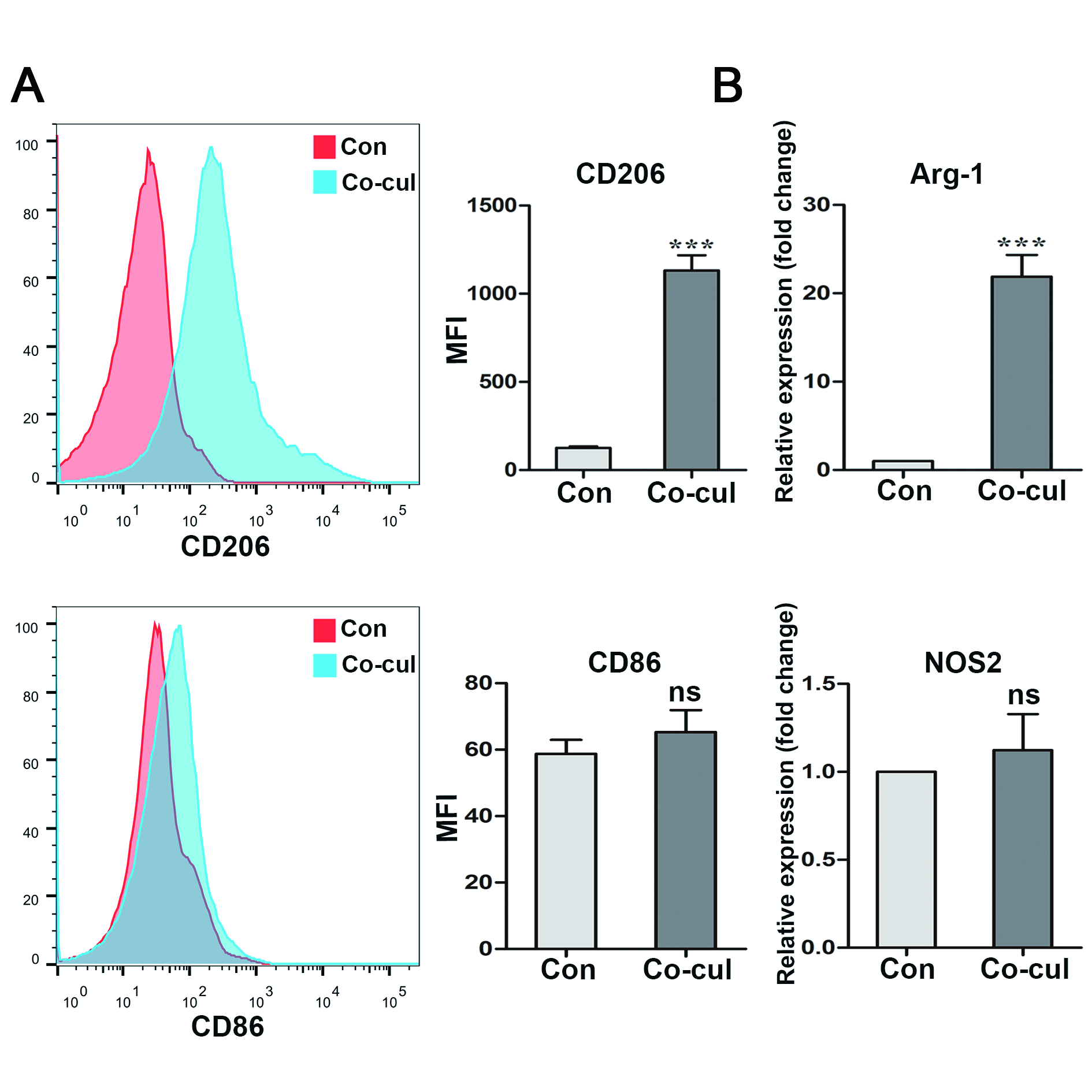


**Supplementary Figure 5. H446 promote NLRP6 expression of THP-1.** Immunoblot analyses of NLRP6 expression in THP-1 co-cultured with H446 (n =3). Con, untreated THP-1. Co-cul, THP-1 co-cultured with H446.


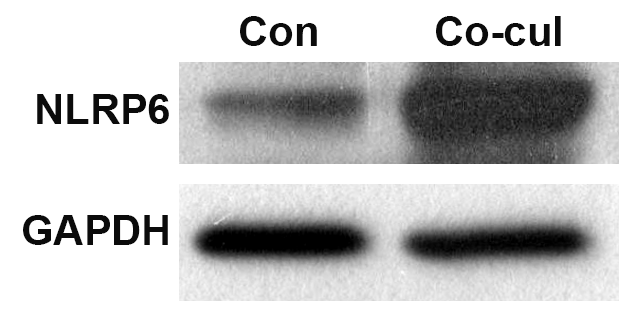


**Supplementary Figure 6. PPAR‐γ and TNF‐α expression level was higher in SCLC-derived exosomes compared with epithelium-derived exosomes.** Immunoblot analyses of PPAR‐γ and TNF‐α expression in SCLC-derived exosomes and epithelium-derived exosomes (n =3). E-Exo, epithelium-derived exosomes. S-Exo, SCLC-derived exosomes.
